# Cycles of myofiber degeneration and regeneration lead to remodeling of the neuromuscular junction in two mammalian models of Duchenne muscular dystrophy

**DOI:** 10.1371/journal.pone.0205926

**Published:** 2018-10-31

**Authors:** Seth G. Haddix, Young il Lee, Joe N. Kornegay, Wesley J. Thompson

**Affiliations:** 1 Texas A&M Institute for Neuroscience, Texas A&M University, College Station, Texas, United States of America; 2 Department of Biology, Texas A&M University, College Station, Texas, United States of America; 3 Department of Veterinary Integrative Biosciences, Texas A&M University, College Station, Texas, United States of America; University of Sydney, AUSTRALIA

## Abstract

Mice lacking the sarcolemmal protein dystrophin, designated *mdx*, have been widely used as a model of Duchenne muscular dystrophy. Dystrophic *mdx* mice as they mature develop notable morphological abnormalities to their neuromuscular junctions, the peripheral cholinergic synapses responsible for activating muscle fibers. Most obviously the acetylcholine receptor aggregates are fragmented into small non-continuous, islands. This contrasts with wild type mice whose acetylcholine receptor aggregates are continuous and pretzel-shaped in appearance. We show here that these abnormalities in *mdx* mice are also present in a canine model of Duchenne muscular dystrophy and provide additional evidence to support the hypothesis that NMJ remodeling occurs due to myofiber degeneration and regeneration. Using a method to investigate synaptic AChR replacement, we show that neuromuscular junction remodeling in *mdx* animals is caused by muscle fiber degeneration and regeneration at the synaptic site and is mimicked by deliberate myofiber injury in wild type mice. Importantly, the innervating motor axon plays a crucial role in directing the remodeling of the neuromuscular junction in dystrophy, as has been recorded in aging and deliberate muscle fiber injury in wild type mice. The remodeling occurs repetitively through the life of the animal and the changes in junctions become greater with age.

## Introduction

Duchene muscular dystrophy (DMD) is a fatal, X-linked recessive disease caused by a mutation in the gene encoding the protein dystrophin [1]. Currently the disease has no known cure. Dystrophin is the key member of a protein complex called the dystrophin glycoprotein complex (DGC) that links the contractile apparatus of muscle fibers to the surrounding extracellular matrix (ECM). The linkage from myofiber to ECM transmits force generated by contraction of the myofibrils laterally [2], and is thought to play a role in protecting the sarcolemma from contraction induced damage [3]. The mutation observed in DMD results in loss of dystrophin expression at the sarcolemma, and the disassociation of the DGC. One theory of dystrophic muscle damage states that the loss of the connection from contractile apparatus to ECM renders DMD muscle fibers increasingly susceptible to contraction-induced injury [4, 5]. Other theories of dystrophic myofiber damage include the DGCs role calcium homeostasis, vasodilation, and inflammation pathways [5]. Following injury, the muscle fiber degenerates locally in the vicinity of damage in a process called segmental necrosis [6, 7]. Segmental necrosis normally is resolved through the action of satellite cells—muscle resident stem cells that fuse to regenerate the damaged portion of the myofiber [6]. Dystrophic fibers are proposed to eventually lose much of their regenerative capacity, possibly due to exhaustion of the number of satellite cells [8]. Lost fibers are replaced with fibrotic and adipose tissue leading to muscle weakness and ultimately death. Another proposed consequence of necrosis and regeneration in this disease is a gross morphological change to the neuromuscular junction (NMJ) as has been shown in aging and deliberately damaged muscles [9–11]. The NMJ is a peripheral synapse that includes the postsynaptic acetylcholine receptor (AChR) aggregate, the innervating motor axon, and the terminal Schwann cells (tSCs) that cap the synapse [12]. It is responsible for elicitation of muscle contractions, so it is not surprising that changes are evident in models of DMD, which is a myopathy. However, a definitive link between fiber necrosis and changes to the NMJ of dystrophic fibers has not been made. Early investigators were able to correlate the incidence of central chains of myonuclei within a myofiber to a remodeled NMJ [9]. These central chains are indicative of myofiber regeneration. In healthy mammalian muscles myonuclei are peripherally positioned near the sarcolemma, and many report that central nuclei will eventually migrate to periphery. The time course for the migration however may be on the scale of months [13]. We have examined two of the most commonly used animal models of DMD, the *mdx* mouse and the canine Golden Retriever Muscular Dystrophy (GRMD) model [14–16]. Both models arose from spontaneous mutations in the dystrophin gene resulting in loss of full-length dystrophin expression. GRMD has a more severe phenotype similar to that of humans. We provide evidence that the morphological changes previously reported in *mdx* NMJs are prevalent in GRMD animals. Moreover, we show that these changes occur early in the life of *mdx* mice and become more frequent and more pronounced into adulthood. We hypothesize that myofiber degeneration and regeneration cause NMJ remodeling in the *mdx* mouse. This study builds on and corroborates evidence that NMJs remodel as muscle fibers regenerate, but does not utilize myonuclear location as a marker for a recent regenerative event. Making use of experimental manipulations possible in mice, we examine the replacement of AChRs at individual junctions in the sternomastoid muscle (STM) and show that replacement is elevated due to myofiber degeneration and regeneration. In contrast to many reports in the literature, we see that myofiber degeneration and regeneration as assessed by receptor replacement is elevated into late adulthood in *mdx* animals. The disease in the STM is not dominated by an early period of necrosis, beginning at about 3 weeks of age, followed by a later period of regeneration and stabilization at 8–12 week weeks of age [17], but occurs at a constant, elevated rate well past the proposed crisis period. Finally, we provide evidence that remodeling of junctions on dystrophic muscle fibers requires the presence of the innervating motor axon and suggest a mechanism for NMJ remodeling—that dystrophic NMJ morphology is initiated by myofiber damage followed by regeneration, and dependent on restructuring cues from the motor axon. If the innervating motor axon is not present remodeling does not occur even following muscle fiber regeneration.

## Materials and methods

### Mouse strains

C57BL/10ScSn-*Dmd*^*md*x^/J mice (*mdx*, RRID:IMSR_JAX:001801) were purchased from Jackson Laboratory and mated with transgenic mice expressing both a green fluorescent reporter expressed in Schwann cells, (B6:D2-Tg(S100B-EGFP)1Wjt/J, RRID:IMSR_JAX:005621) and a cyan fluorescent reporter expressed in motor axons (B6.Cg-Tg(Thy1-CFP)23Jrs/J, RRID:IMSR_JAX:003710). Use of these mice has been previously reported and all mice are available from Jackson Laboratories. *mdx* mice were crossed into fluorescent mice, and progeny back crossed into *mdx* for 8 generations to ensure a C57BL/10 isogenetic background in fluorescent *mdx* mice. 4–7 male mice per genotype and age were used for each experiment, for a total of 59 mice. Experimental procedures in the Formation and maintenance of neuromuscular synapses (2016–0158) protocol were approved by Texas A&M University IACUC.

### In vivo two-color α-bungarotoxin

Mice were anesthetized via isoflurane inhalation and placed in a supine position. Neck hair was removed using a depilatory and the surgical site sterilized with povidone-iodine. An incision was made from chin to sternum and the skin retracted. The salivary glands were moved to the side to expose the STM. Surrounding connective tissue was gently removed and a non-saturating concentration (diluted 2 μg/mL in sterile lactated Ringers) of α-bungarotoxin conjugated to Alexa Fluor^™^ 555 (ThermoFisher Scientific, Cat# 35451) applied to the surface of the muscles for 5 minutes (BTX-1). The neck cavity was then washed copiously with lactated Ringer’s ten times. For muscle injury experiments, superficial muscle fibers at areas close to the site of entry of the accessory nerve into the muscle were cut with a sterile #15 surgical scalpel. The contralateral muscle was also labeled with BTX-1, but not injured and used as an internal control (IC). For denervation experiments both STM muscles were labeled with BTX-1 and the accessory nerve of one muscle was severed near its insertion site into the muscle. The other muscle was spared and used as an IC. The salivary glands were moved back into place following labeling, washing, and injury. The skin was sutured with 8–0 silk and treated with 2% lidocaine hydrochloride jelly. Animals were allowed to convalesce for ten days post-surgery. Endpoints for the studies are labeled P38, P66, P160, and P450. P38 animals were 38 ± 3 days old, P66 animals were 66 ± 3 days old, P160 animals were P160 ± 3 days old, and P450 animals over 450 days old at the time of death. Mice were killed via I.P. injection of 0.15 mL of Euthasol. The STM was dissected, pinned at resting length to a sylgard dish and fixed using 4% phosphate buffered paraformaldehyde (PFA) for 20 minutes. After fixation the muscles were washed three times with phosphate buffered saline (PBS) and labeled with α-bungarotoxin conjugated to Alexa Fluor^™^ 647 (ThermoFisher Scientific, Cat# 35450) for 5 minutes (BTX-2, diluted 2 μg/mL in PBS). DAPI (Thermo Fisher Scientific Cat# D3571, RRID:AB_2307445) was used to label nuclei (diluted 0.5 μg/mL in PBS). After three PBS washes, a longitudinal “filet” from across the surface of the muscle was dissected from distal tendon to proximal tendon and mounted on a microscope slide in anti-fade fluorescence mounting medium.

### Image acquisition, and NMJ characterization and analysis

Junctions were counted and categorized using a Leica DMRX epifluorescence microscope and a 40X Oil objective (NA 1.4). Junctions were considered fragmented if they had 5 or more non-connected clusters of AChRs in close proximity to each other on the same myofiber. Junctions were categorized based of the brightness of BTX-1 and BTX-2 labels, as well as if they were continuous (< 5 AChR clusters), or fragmented (> 5 AChR clusters). They were classified as stable if BTX-1 and BTX-2 labels were both bright, or as dynamic if BTX-1 label was totally or mostly absent and BTX-2 label bright. In many cases dynamic junctions did not completely lose BTX-1, and small puncta of fluorescence was seen. It is likely that these puncta are BTX-1 labeled receptors tethered to the basal lamina and demarcate the initial synaptic site before myofiber degeneration. If both the BTX-1 and BTX-2 labels were faint, junctions were classified as lost, but this was rarely observed. In order to verify that dynamic junctions indeed lose their BTX-1 label, a subset of STMs were removed immediately following the initial exposure, at Day 0 without any convalescence. After dissection the Day 0 muscles were labeled with BTX-2. In all junctions BTX-1 and BTX-2 labels were bright and junctions were all categorized as stable. Characterization and counts were performed by multiple researchers on the same muscle preparation, and similar results obtained. 50 to 100 NMJs were characterized for each muscle preparation.

Confocal micrographs were collected on either a Leica TPS II SP5 or a Zeiss LSM780 microscope using a 40X Oil objective (NA 1.4). Step size was set between 300–500 nm. Laser lines 405 nm, 458 nm, 488 nm, 563 nm, and 633 nm were used as necessary. Images were analyzed in ImageJ (ImageJ, RRID:SCR_003070) by creating maximum intensity projection images from collected stacks. Junctional Area was calculated by thresholding the BTX-2 labeled channel using the Auto Threshold tool provided by ImageJ. A polygon shaped region of interest was then circumscribed around the thresholded signal and the region of interest’s area measured. Receptor Area was measured as the thresholded area of the BTX label within the circumscribed region of the AChR. Dispersion Index [11] was calculated as Receptor Area/Junctional Area. The number of AChR fragments (Fragmentation) was measured using the objects counter in ImageJ by setting the minimum area for counts as 5 μm^2^. While this measurement does not consider fragments in separate z-planes, as they may be superimposed in our maximum intensity projections, it is a conservative estimate of fragmentation. The actual number of fragments for both WT and *mdx* is likely underestimated by this measurement. tSC counts were made by counting only fluorescently labeled Schwann cells in proximity to the endplate having coincident nuclear label via DAPI. Axon branch points were measured by creating 3D rotations of the confocal images of fluorescently labeled motor neurons, and then counting the number of times the axon splits. Small varicosities of the motor terminal were not counted as branches.

### Canine use

Dogs were used and cared for in accordance with the National Research Council’s Guide for the Care and Use of Laboratory Animals and housed at Texas A&M University. Procedures used were approved by university IACUC protocol, Standard Operating Procedures-Canine X-Linked Muscular Dystrophy (2012–052 or 2015–0110). Ages of dogs ranged from 1 to 6 years. 3 male wild-type (WT) controls and 7 male GRMD dogs were used, for a total of 10 animals. The animals used were undergoing necropsy for other purposes and the tissues shared. These dogs were part of a colony owned and maintained by Texas A&M University used for research purposes.

### Canine muscle preparations

Following euthanasia for non-related procedures via I.V. injection of Euthasol, the middle portion of cranial tibial muscles was dissected and placed in 4% PFA for 20 minutes. After fixation, the muscles were washed three times with PBS. Muscle fiber fascicles were dissected from the section and labeled with BTX conjugated to Alexa Fluor^™^ 555 overnight in PBS (diluted 2 μg/mL). Labeled fascicles were washed in PBS three times and then examined under a fluorescence dissecting microscope to identify the endplate band. A small portion of the fascicle containing the endplate band was cut from the fascicle and embedded in a 3% agarose gel. 40 μm longitudinal sections (parallel to the long axis of muscle fibers) were prepared using a vibratome. These sections were blocked using standard blocking solution (0.2% bovine serum albumin, 0.3% Triton 100X, 0.1% sodium azide in PBS) and labeled via mouse monocolonal antibodies to neurofilament (SMI-31, Covance, RRID:AB_531793, diluted 4 μg/mL in standard blocking solution), synaptic vesicles (DSHB Cat# SV2, RRID:AB_2315387, diluted 4 μg/mL in standard blocking solution) and rabbit polyclonal antibodies to Schwann cells (S100, Dako Cat# Ga504, diluted 4 μg/mL in standard blocking solution) in PBS overnight. After three PBS washes, goat anti-rabbit secondary antibodies conjugated to AlexaFluor^™^ 647 (Thermo Fisher Scientific Cat# A-21245, RRID:AB_2535813), and goat anti-mouse secondary antibodies conjugated to AlexaFluor^™^ 488 (Thermo Fisher Scientific Cat# A-11001, RRID:AB_2534069) were applied for two hours in PBS (diluted 2.5 μg/mL in standard blocking solution), along with BTX conjugated to AlexaFluor^™^ 555 (diluted 2 μg/mL in standard blocking solution). DAPI was used to label nuclei (diluted 0.5 μg/mL in standard blocking solution). Following three additional PBS washes, the sections were mounted on microscope slides using an anti-fade mounting medium.

### Statistics

#### Mice

For analyses of mouse confocal images, STMs from 3–5 individuals of each group were used. Animals were grouped by approximate and genotype (P38 WT, P38 *mdx*, etc). 5–20 NMJs were imaged per muscle. NMJs were pooled for statistical analysis of confocal measurements. P38 animals were 38 ± 3 days old, P66 animals were 66 ± 3 days old, P160 animals were P160 ± 3 days old, and P450 animals over 450 days old at the time of death. Measurements were averaged and a Student’s t-test preformed to assess for significant differences between genotypes. Within genotypes but among ages, 2-Way ANOVA with Bonferroni post-hoc tests were used. The degrees of freedom for each measurement is reported in text as t(df) = t-value, P-value. For t-tests performed on these measurements the sample size n (number of pooled individual NMJs) can be calculated as df + 2.

For NMJ categorization (e.g. stable, continuous; dynamic, fragmented…), 4–7 animals per genotype and age were used. 50–100 junctions were categorized per muscle, based on the brightness of the BTX-1 label (e.g. stable, dynamic) and the morphology of the BTX-2 label (e.g. continuous, fragmented, or lost). Lost junctions are considered dynamic. Category counts were then transformed into percentages of total counted NMJs for each muscle prep. These percentages were averaged between groups (P38 WT, P66 *mdx*…). 2-Way ANOVA with Bonferroni post-hoc analyses were performed to assess significant differences both between genotypes and within genotypes. The degrees of freedom for each measurement is stated in text as t(df) = t-value, P-value. For statistical tests performed on these measurements, the sample size n (the number of mice compared) can be calculated as df + 2. Statistical analysis was performed using Prism GraphPad 5 software (GraphPad Prism, RRID:SCR_002798). Statistical significance was set at P < 0.05. In figures and text, results are presented as means with SEM unless otherwise noted.

#### Dogs

For measurements of dog confocal images, muscle preparations 3 WT controls and 7 GRMD dogs were used, for a total of 10 animals. 20–50 NMJs were imaged per individual and measurements were made as described above in “Image acquisition and analysis”. These measurements were pooled by genotype. A Student’s t-test was performed between genotypes on averages of each measurement. The degrees of freedom for each measurement is reported in text as t(df) = t-value, P-value. For t-tests performed on these measurements the sample size n (number of pooled individual NMJs compared) can be calculated as df + 2. Statistical analysis was performed using Prism GraphPad 5 software, and statistical significance was set at P < 0.05. In figures and text, results are presented as means with SEM unless otherwise noted.

## Results

### *mdx* mice undergo progressive fragmentation of their AChR rich endplate

**Fig 1:**
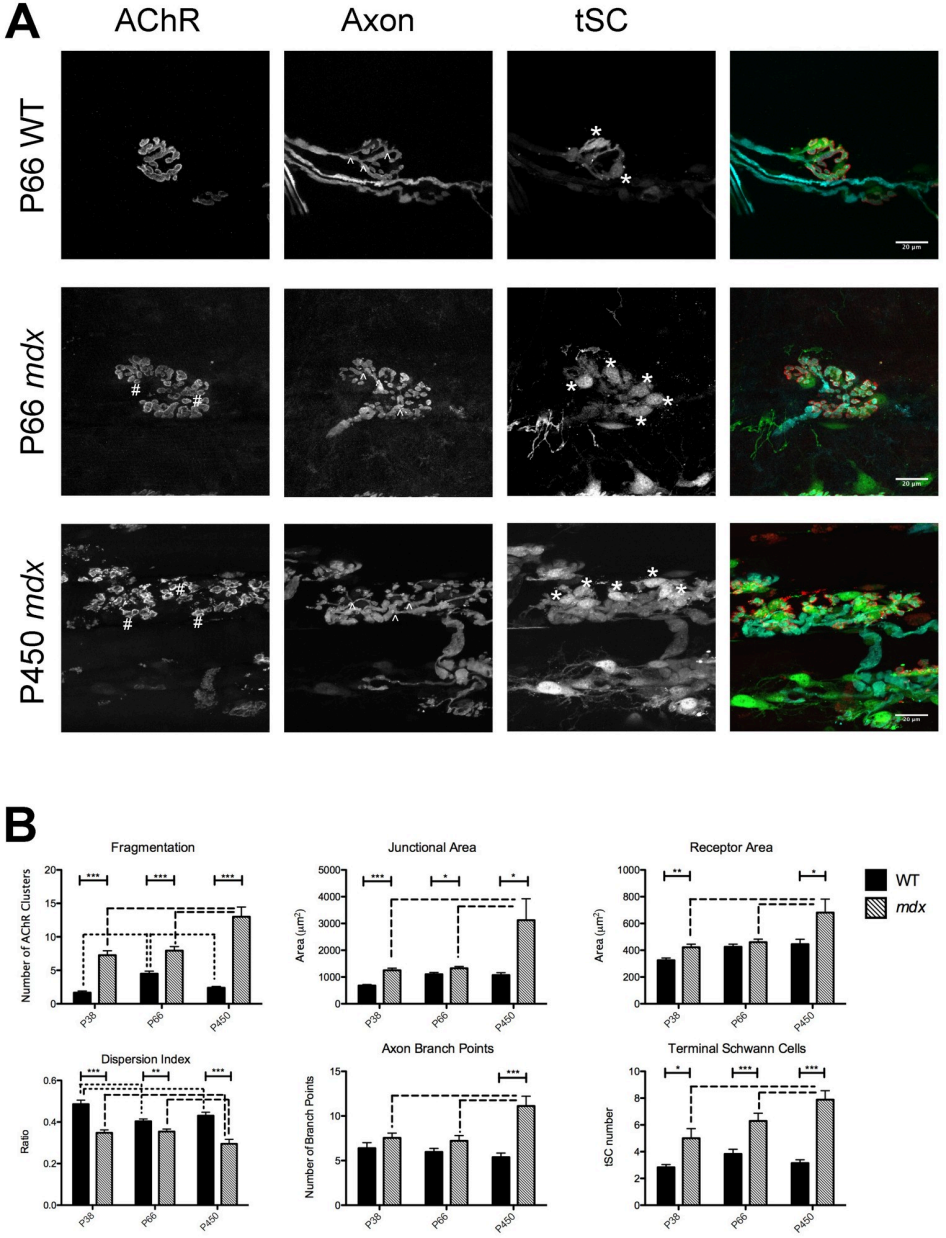
WT and *mdx* NMJs. (A) Maximum intensity projection confocal images of P66 WT, P66 *mdx* and P450 *mdx* NMJs. Images from left to right: AChR, motor axon terminal arbor, tSCs, and composite images. In the composite image, AChRs are pseudocolored red, motor axons cyan, and tSCs green. Scale bar = 20 μm. “#” indicates examples of AChR fragments; arrowheads, examples of axon terminal branch points; asterisks cell bodies of tSCs. In P450 *mdx*, not all tSCs are marked. Some non-specific labeling via the S100 promoter of cells that cannot be positively identified as Schwann cells is seen. These cells are not labeled as tSCs in the panels nor included in measurements. (B) Junctional measurements in WT and *mdx* NMJs from P38 to P450 as described in “Image acquisition and analysis”. Asterisks above solid bars denote significance between genotypes (t-test, * P < 0.05, ** P < 0.001, *** P < 0.0001). 15 to 90 NMJs were compared for each measurement. Exact n can be found in text as t(df) = t-value. n = df + 2. n is pooled number of individual NMJs compared. Dashed lines denote significance between ages (2-Way ANOVA, Bonferroni post-hoc test, P < 0.05).

Using fluorescence confocal microscopy, we found changes to NMJ morphology in *mdx* mice similar to those reported previously [9–11]. We imaged *mdx* muscles near the onset of necrosis (P38) and onward and these changes were present in the youngest animals examined, and older adult *mdx* animals show morphological differences to the younger *mdx* mice indicating disease progression. When labeled with fluorescently tagged α-bungarotoxin (BTX), a highly specific ligand for the AChRs, the receptors at most individual *mdx* NMJs were fragmented into discontinuous aggregates. This differs from the pretzel-like receptor aggregates of WT animals, which are arranged into continuous branches (Fig 1A). Junctions with this abnormal clustering of AChRs have been classified as fragmented into “islands” [9, 11, 18]. Maximum intensity projections of confocal stacks were evaluated for the number of AChR fragments they contained. *mdx* NMJs had more fragments than WT at all ages (Fig 1B. Fragmentation: 336% increase at P38 t(32) = 7.974, P < 0.0001, 77% increase at P66 t(75) = 4.756, P < 0.0001, and 445% increase at P450, t(19) = 7.186, P < 0.0001, t-tests). Notably, at P38 in *mdx* there was a population of both fragmented (56.7%) and continuous junctions. We think it likely, as others have postulated [10], that dystrophic junctions remodel from continuous to fragmented as the animals age, that this remodeling is stochastic in its occurrence, and that this process is irreversible. The irreversibility is suggested by two observations. First, the proportion of junctions that are fragmented increases as *mdx* mice age and does not fall. At early time points (P14), WT and *mdx* animals have roughly the same proportion of adult-like, continuous junctions [10] but from P66 on nearly all the AChR aggregates are fragmented in appearance in *mdx*. Additionally, the degree of fragmentation becomes more severe as dystrophic animals age, with the number of fragments increasing from 7.3 in P38 *mdx* animals to 13.0 in P450 *mdx* animals (Fig 1. t(90) = 5.460, P < 0.001, 2-Way ANOVA, Bonferonni post-hoc test). In WT animals no such increase from P38 to P450 was evident, and the average number of fragments at all ages was less than five.

### *mdx* mice undergo progressive NMJ expansion and a dispersion of AChRs within the synaptic area

Fragmentation is one of the most striking postsynaptic changes in *mdx* NMJs, but other measurable differences are also evident. In order to approximate the size of the entire synaptic structure (i.e. the AChR aggregate, the motor axon terminal, and the tSCs) a polygon was drawn around the labeled receptor aggregate and its area was measured. This polygon included the totality of the receptors but also included areas between the receptor aggregates that were not labeled with BTX. Thus, the polygon included areas containing tSC somata and processes, and the motor axon terminals ramifying above and between the synaptic contacts, even if these entities were not fluorescently labeled or imaged. We designate this measurement the Junctional Area. At all ages, the Junctional Area was larger in *mdx* than in WT (Fig 1B. P38 t(36) = 6.581, P < 0.0001, P66 t(84) = 2.319, P = 0.0228, P450 t(19) = 2.537 P = 0.0201, t-tests). WT Junctional Area ranged from ~35% to ~80% of the *mdx* Junctional Area.

There was also an increase in Junctional Area in *mdx* from P38 to P450 (Fig 1B, t(90) = 5.089, P < 0.001, 2-Way ANOVA, Bonferroni post-hoc test), and P66 to P450 (Fig 1B, t(90) = 5.380, P < 0.001, 2-Way ANOVA, Bonferroni post-hoc test) that was not noted in WT animals. P450 *mdx* Junctional Area was about 2.5x that of P38 *mdx*.

An effort was made to measure just the area that AChRs occupied on the sarcolemma, not including the motor axon and tSCs. The Receptor Area was defined as the area within the Junctional Area that fluoresced intensely when labeled with BTX conjugated to an AlexaFluor^™^ marker. *mdx* displayed increased Receptor Area from WT only at P38 and P450 (Fig 1B. P38, t(41) = 3.284, P = 0.0021 and P450, t(24) = 2.195 P = 0.0381, t-tests). At P38 *mdx* Receptor Area was 1.3x as large as P38 WT, while at P450 *mdx* Receptor Area was 1.5x as large as P450 WT. In WT endplates, there were no significant changes in Receptor Area throughout life. Interestingly the Receptor Area in *mdx* increased from ages P38 to P450 (Fig 1B. t(90) = 4.277, P < 0.001, 2-way ANOVA, Bonferroni post-hoc test). There was no increase in Receptor Area from P38 to P66 in *mdx*. It should be noted that Receptor Area is not a receptor density measurement. While the space the receptors occupy on the myofiber increases, the density may or may not change at the same time. We chose not to rely on fluorescence intensity measurements to estimate receptor density as this measurement varies with junctional position in the muscle preparation thickness.

We also investigated the spread of receptors within the synaptic area. To quantify this spread, the Receptor Area was divided by the Junctional Area and termed the Dispersion Index (DI), a measure previously used by Pratt and colleagues [11]. The lower this ratio the more spread there is of the receptor clusters within the junction. Junctions in the *mdx* animals were consistently more disperse, i.e. had a lower DI than age matched WT (Fig 1B. P38 t(34) = 5.926 P < 0.0001, P66 t(88) = 3.117, P = 0.0025, P450 t(38) = 5.034, P < 0.0001, t-tests). The DIs of *mdx* animals were ~70%-90% of WT. Interestingly the spread of the receptors within the junction at P450 *mdx* is 1.2x that of P38 *mdx* animals (t(90) = 2.264, P < 0.05, 2-Way ANOVA, Bonferroni post-hoc test). As with Fragmentation, this suggests that as *mdx* animals age an increasing number of NMJs remodel from the healthy continuous state to the large, fragmented dystrophic state. The results also suggest that individual NMJs can become increasingly remodeled with time. By P66 nearly all the *mdx* NMJs are remodeled, but significant differences from P66 to P450 can be seen in all the postsynaptic characteristics measured in *mdx*. It is likely that ongoing degeneration and regeneration of the STM myofibers is driving these progressive alterations.

### *mdx* NMJs undergo a remodeling of the presynaptic apparatus

It has been established that *mdx* mice undergo bouts of myofiber necrosis and regeneration due to their high susceptibility to myofiber damage. Correspondingly, muscle fiber damage has been shown to rearrange the NMJ. Based on observations in vivo of deliberate muscle fiber damage, this is thought to occur through the motor axon terminal, which is normally directly apposed to the receptor islands that appear after regeneration of each damaged fiber. The motor terminal was found to extend processes along the regenerating muscle fiber and it was proposed that the motor neuron directs the insertion of AChRs into new contact sites in the sarcolemma, resulting in a larger and fragmented endplate with a more complex motor axon terminal arbor [19]. The motor terminal was found to extend processes along the regenerating muscle fiber and it was proposed that the motor neuron directs the insertion of AChRs into new contact sites in the sarcolemma, resulting in a larger and fragmented endplate with a more complex motor axon terminal arbor. It is probable this phenomenon is occurring in dystrophic mice as well. To investigate this possibility, measurements on the presynaptic apparatus of NMJs, namely the tSCs and motor axon terminal, were performed. *mdx* animals were crossed to transgenic mice that express eGFP driven by the S100 promoter in Schwann cells [20], and CFP in motor axons driven by the Thy1 promoter [21]. The number of motor axon branch points in proximity to the endplate was considered a measure of motor neuron terminal rearrangement. Branching pattern differences between *mdx* and WT animals were not significant except at P450 (Fig 1B. Branch Points, t(21) = 4.816 P < 0.0001, t-test). It was noted, however, that the gross morphology of dystrophic terminal arbors was visually distinct from WT. As the motor neuron contacts the muscle fiber at the endplate of healthy WT animals, branches of the terminal tend to run smoothly along the tracks formed by the AChR gutters. *mdx* axonal arbors were seen to have small, bulbous varicosities that made connections to the fragmented islands of AChRs (Fig 1A). These varicosities were not counted as branches because they are very short. The number of axonal branches did increase from 7.5 to 11.1 (Fig 1B. t(52) = 4.010, P < 0.001, 2-way ANOVA, Bonferroni post-hoc test) in *mdx* mice from P38 to P450. This trend was not apparent in WT mice, consistent with previous reports that normal junctions can grow and shrink without changing their branching pattern [22].

The number of tSCs was counted for both *mdx* and WT junctions based on GFP expressed in these cells and coincident nuclear DAPI label (S1 Fig). Dystrophic endplates consistently had more tSCs than age matched WT endplates (Fig 1A and 1B. Terminal Schwann Cells, P38, t(8) = 2.880 P = 0.0205, P66 t(33) = 3.655, P = 0.009, P450 t(21) = 6.711, P < 0.0001, t-tests). In *mdx* the number of tSCs increased with age, from P38 to P450 (Fig 1B. t(45) = 3.441, P < 0.01, 2-Way ANOVA, Bonferroni post-hoc test). The phenomenon was not apparent in WT mice. On average WT P38 animals had 2.8 tSCs per NMJ, while P450 had 3.1. In contrast, at the same ages, *mdx* NMJs had on average 5.0 tSCs at P38 and 7.9 at P450. These findings fit well with observations that tSCs are added to the NMJ during the process of junction growth during development [23]. During periods of little to no growth in healthy adult mice no tSCs are added. However, during the expansion of the endplate from P38 to P450 in *mdx* mice, as suggested by increased Junctional Area, there is also an expected addition of tSCs.

### GRMD dogs display similar changes in NMJ morphology to those in *mdx* mice

**Fig 2:**
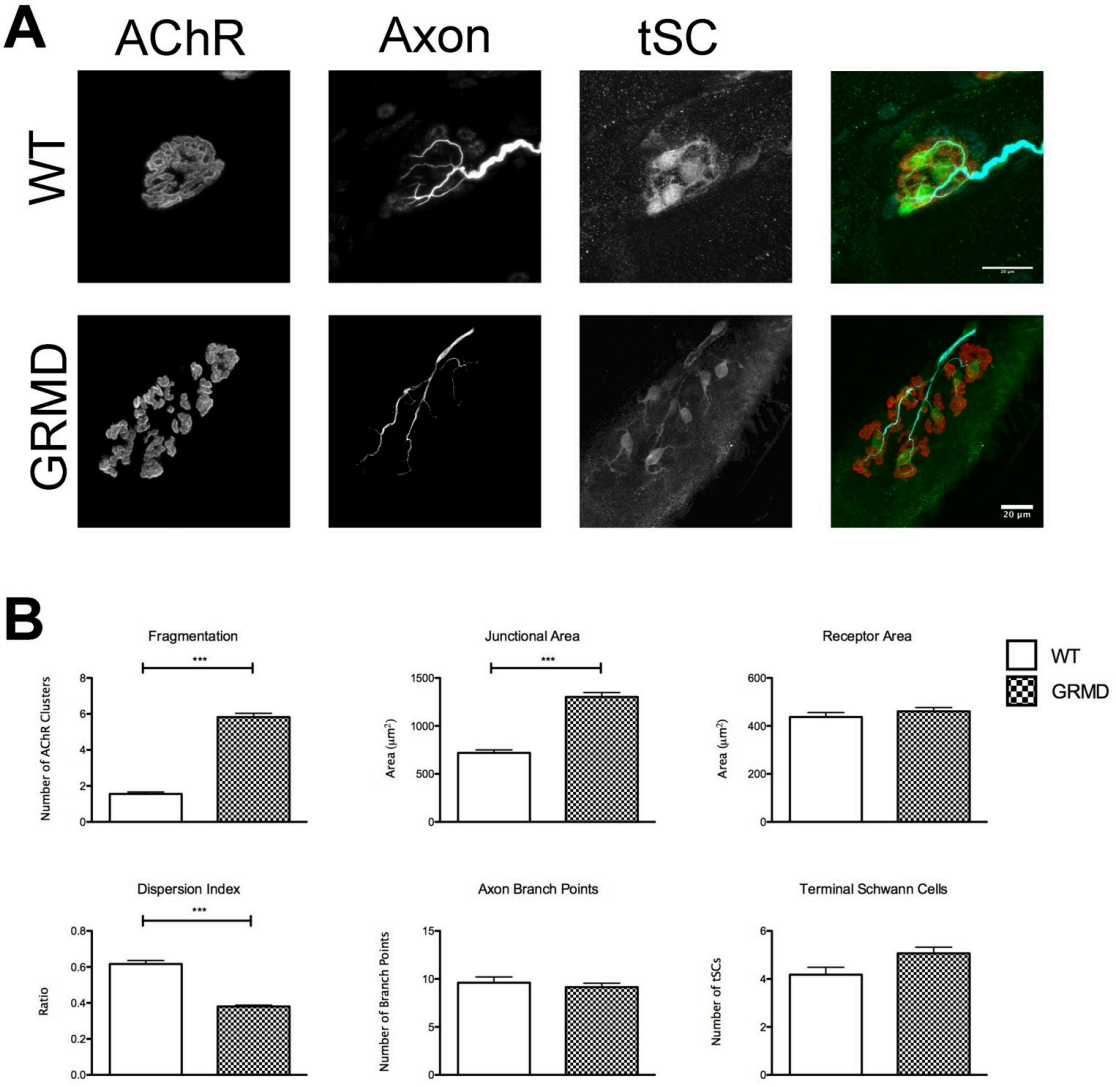
GRMD NMJ morphology. (A) Confocal maximum intensity projection of adult WT and GRMD NMJs harvested from the cranial tibial muscle. Composite images pseudocolored red-AChR, cyan-motor axon, green-tSC. Scale bar = 20 μm. Note the difference in magnification between WT and GRMD. (B) Junctional measurements in WT and GRMD NMJs as described in “Image acquisition and analysis”. Asterisks denote significant differences (t-test, P < 0.0001). Exact n can be found in text as t(df) = t-value. n = df + 2. n is number of pooled individual NMJs compared.

*mdx* mice have a mild phenotype and near-normal lifespan compared to humans afflicted with DMD. Therapies that are effective in treating dystrophic mice have not translated well to humans in clinical settings [24, 25]. We, therefore, sought to determine if the morphological changes seen in *mdx* mice also occur in GRMD, a canine model more clinically relevant to DMD [26]. We harvested the midsection of cranial tibial muscles in the pelvic limb of adult GRMD and WT dogs at necropsy performed for other non-related experiments. AChRs, Schwann cells, and motor neurons were fluorescently labeled via immunofluorescence and BTX. When imaged using confocal microscopy, changes to the NMJ like those in *mdx* mice were found. The postsynaptic AChR aggregate, labeled via fluorescent BTX, was clearly abnormal (Fig 2A). The same NMJ measurements made on murine synapses were also made for canine preparations. The NMJs of GRMD dogs were 1.8x as large as their WT counterparts (Fig 2B. Junctional Area, t(383) = 6.627, P < 0.0001, t-test). The spread of receptors in GRMD NMJs is 1.6x that of WT (Fig 2B. Dispersion Index, t(383) = 13.72, P < 0.0001, t-test). Finally, the number of fragments of AChRs was higher in dystrophic dogs than in WT (Fig 2B. Fragmentation, t(383) = 10.59, P < 0.0001, t-test). WT animals had an average of 1.6 fragments per NMJ, and GRMD 5.8. No significant difference was seen in the Receptor Area of the two groups. This result is similar to P66 mice and may represent a homeostatic event in affected animals that compensates for remodeling of their NMJs at least at the ages examined.

When the presynaptic apparatus of the NMJ was investigated, no differences in the number of motor axon branches were noted (Fig 2A and 2B, t-test), similar to the case of younger *mdx* reported above. In some preparations, it was evident that GRMD axon terminals also synapsed on the myofiber in varicosities (S2 Fig) similar to that of *mdx*. However, this was not apparent in all images, and is likely due to incomplete labeling using the antibodies to synaptic vesicles and neurofilament. Consistently weaker labeling was observed in the canine preparations than those obtained in mice. It is possible that older GRMD dogs would show increased changes like those in the P450 *mdx* mice, but this was not investigated. Finally, there was no difference in the number of tSCs between GRMD and WT dogs, but again immunofluorescent labeling was the method used in dog preparations. In the mouse investigations, the use of transgenic fluorescent proteins in the Schwann cells and the motor neurons allowed for more faithful labeling. Regardless, the similarities between *mdx* and GRMD NMJs suggest that remodeling occurs in all mammals that lack dystrophin. This includes boys with DMD. What exactly is causing these changes has not been satisfactorily investigated, however. Using the *mdx* mouse, we were able to investigate if myofiber degeneration and regeneration were indeed causative in remodeling.

### AChR replacement is elevated in *mdx* mice throughout life

**Fig 3:**
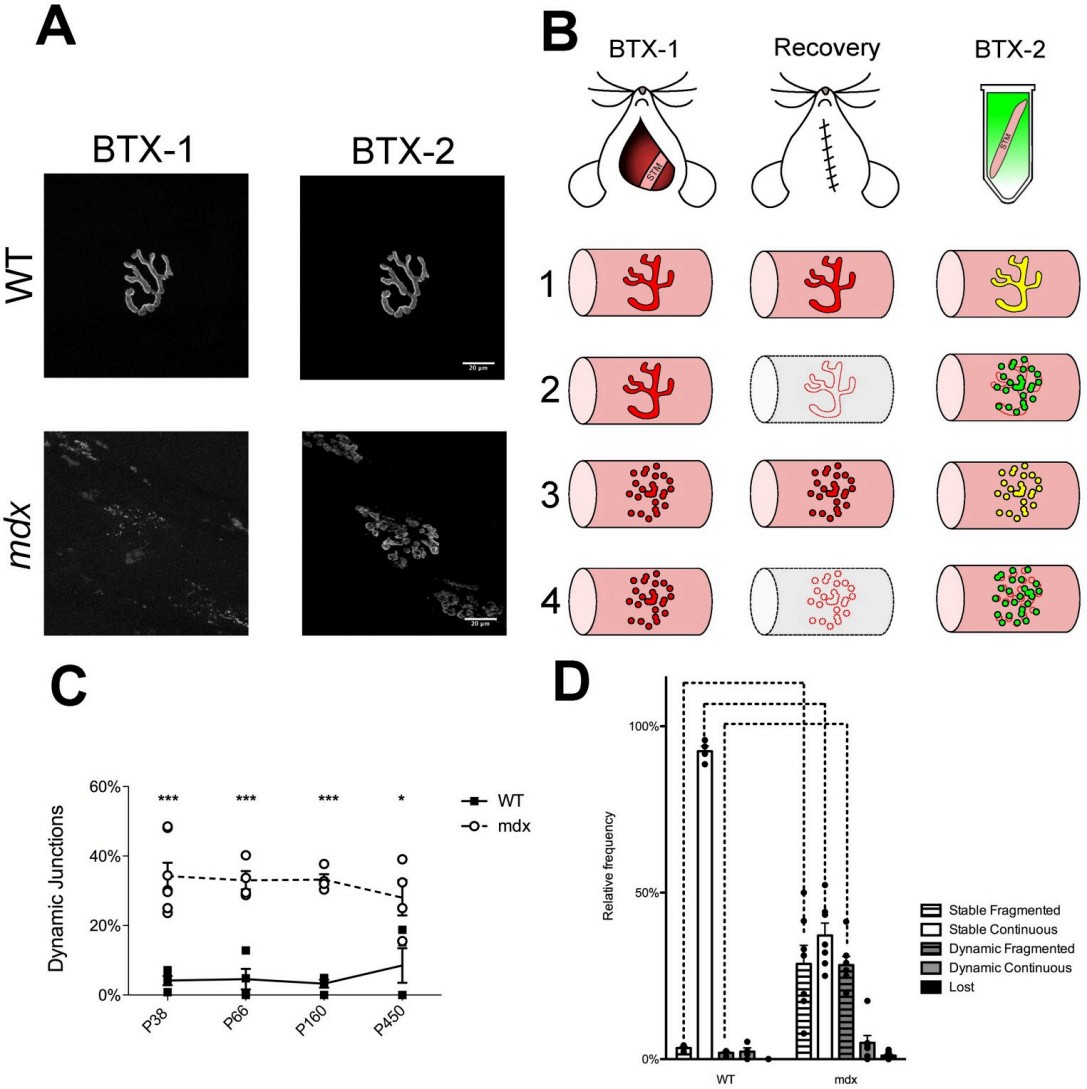
Receptor replacement and NMJ categorization following two-color BTX. (A) Maximum intensity projection images of STM whole mounts that have undergone the two-color BTX procedure. Examples are from P66 mice. BTX-1 is the first receptor label. BTX-2 is the second applied 10 days later. Imaging follows muscle dissection. In *mdx* there is a loss of BTX-1 and frequently the BTX-2 label shows fragmentation. Scale bar = 20 μm (B) Two-color BTX experimental design. The STM of mice was exposed and labeled with BTX-1 at Day 0. The animal was allowed to recover from the surgery for 10 days and then euthanized. The labeled muscle was dissected from the animal and labeled with BTX-2. The lower portion of (B) illustrates four predicted outcomes of the experiment. In red is a possible morphology of BTX-1 labeled AChR aggregates at Day 0 of the experiment, in green BTX-2 label at Day 10. Colocalization of BTX-1 and BTX-2 is indicated with yellow. Intact myofibers are shown as pink tubes, and a degenerating myofiber is shown as a gray tube. Scenario **1** shows a stable, continuous junction. **2** a dynamic, fragmented junction. **3** a stable fragmented junction, and **4** another dynamic, fragmented junction. (C) Receptor replacement in WT and *mdx* STM. Receptor replacement, calculated as percentage of dynamic junctions, in *mdx* mice is constantly elevated compared to WT. Asterisks denote significance (* P < 0.05, *** P < 0.001, 2-Way ANOVA, Bonferroni post-hoc test). No effect of age was found on receptor replacement. (D) Categorization of P38 WT and *mdx* junctions following two-color BTX. Bars below labels denote significant difference within genotypes. Not all significant differences within genotypes are shown for sake of brevity. Dashed lines above graph denote significance between genotypes (2-Way ANOVA, Bonferroni post-hoc test, P < 0.05). A full account of statistical significance can be found in S1 Table. Exact n can be found in text as t(df) = t-value. n = df + 2. n is number of mice compared.

We investigated whether the dynamics of the AChRs could provide evidence of how the NMJ becomes remodeled. The rationale behind the approach is that loss of receptors from the myofiber membrane should indicate recent degeneration of the myofiber [27]. As the muscle fiber undergoes necrosis, the AChRs on its surface would also be destroyed during breakdown of the sarcolemma. Upon subsequent regeneration of the myofiber, new AChRs would have to be inserted into the membrane to allow the resumption of neuromuscular transmission. If a loss of receptors is coincident with abnormal endplate morphology, this would suggest that necrosis and regeneration at the endplate spurs morphological changes to the NMJ. To investigate this possibility, the AChRs of STMs were labeled with a sub-saturating dose of fluorescently tagged BTX (BTX-1) in live *mdx* and WT mice (Fig 3B). The concentration and exposure time have been shown previously to not affect neuromuscular transmission or NMJ morphology [28]. Following a 10-day recovery period (endpoints of P38, P66, P160, and P450) that allows for the occurrence of any ongoing myofiber degeneration and regeneration, the labeled muscle was dissected from the animal and labeled with a second spectral variant of fluorescently tagged BTX (BTX-2). This method is termed the in vivo two-color BTX method. Using epifluorescence microscopy, the junctions were then categorized based on both their morphology (fragmented or continuous) and the presence of each of the two spectral labels. If BTX-1 was lost and BTX-2 was bright, junctions were categorized as dynamic. If the brightness of the BTX-1 label was similar to BTX-2, junctions were categorized as stable (Fig 3A and 3B). If both BTX-1 and BTX-2 were weak, the junction was categorized as lost. It has been noted that there is rarely a complete removal of the BTX-1 label even after a bout of myofiber degeneration that follows deliberate damage to fibers. This residual label appears punctate and probably marks the initial synaptic site where some receptors remain tethered to the basal lamina after myofiber degradation [19, 29]. S3 and S4 Figs show a number of examples of categorization, indicate the reproducibility of this technique, and show the stochastic nature of BTX-1 loss. In WT mice the frequency of dynamic junctions is consistently low from P38 to P450, indicating no dramatic loss of receptors over the 10-day interval. However, when *mdx* animals were investigated, the proportion of dynamic junctions was 3.3x to 10x as large as WT controls at P38 to P450 (Fig 3A. P38 t(9) = 6.468, P < 0.001, P66 t(6) = 5.433, P < 0.001, P160 t(6) = 5.712, P < 0.001, P450 (6) = 3.719, P < 0.01, 2-Way ANOVA, Bonferroni post-hoc test). Lost junctions were considered dynamic in these measurements, as they also indicate degeneration of the myofiber. Surprisingly, there was no significant effect of age on the frequency of dynamic junctions in *mdx* muscles, indicating that receptor replacement in the STM is not confined to an early period during the lifetime of *mdx* mice but occurs constantly from P38 to P450 (Fig 3A). While dynamic junctions were used in this report to measure myofiber degeneration and regeneration, there was some evidence of central chains of nuclei directly under dynamic, fragmented junctions (S1 Movie).

When the morphology of junctions was assessed between *mdx* and WT animals, we noticed that the predicted decrease in the number of stable, continuous junctions in *mdx* (Fig 3D. t(9) = 12.10, P < 0.001, 2-Way ANOVA, Bonferroni post-hoc test) was accompanied by an increase in the number of dynamic, fragmented junctions (Fig 3D. t(9) = 5.763, P<0.001, 2-Way ANOVA, Bonferonni post-hoc test). This suggests that junctions shift from continuous to fragmented during muscle regeneration. P38 muscles were used in this investigation, as after this time point a great majority of *mdx* endplates are fragmented, and once fragmented NMJs have not been shown to revert back to a continuous morphology. In P38 *mdx* animals, the two NMJ morphologies present allowed us to assess whether or not receptor replacement has any bearing on NMJ morphology. When the morphologies of dynamic junctions were compared within P38 *mdx* animals dynamic, fragmented junctions were seen at a higher frequency than dynamic, continuous (Fig 3D. t(30) = 5.992, P < 0.001, 2-Way ANOVA, Bonferroni post-hoc test). Again, this suggests that in *mdx* animals, AChR replacement is associated with NMJ remodeling. This correlation was not apparent in P38 WT animals. In WT animals, we found a small fraction of dynamic junctions (both fragmented and continuous) with no significant difference between their morphologies (Fig 3D). While we predicted that in both WT and *mdx* mice dynamic junctions would preferentially be fragmented, the WT observation that receptor replacement had no effect on junction morphology may be a result of the overall low levels of fiber damage even in normal muscles. As such, any striking differences in the morphology of dynamic junctions might have been masked. While not indicated in Fig 3 for sake of brevity, a full account of NMJ categorization differences (i.e. dynamic, fragmented vs stable, fragmented) is presented in S1 Table. Overall, these results suggest that cycles of myofiber degeneration and regeneration lead to remodeling of their junctions in dystrophy. An alternative explanation is that more rapid receptor replacement is a characteristic of *mdx* junctions because dystrophin tethers receptors to the membrane as suggested from gamma counting of radio labeled AChRs in whole muscles [30]. This is unlikely given the presence of both dynamic and stable junctions in the same muscle. Elevated receptor replacement was encountered at only a subset of junctions. In fact, dynamic junctions were observed on fibers neighboring those with stable junctions. If dystrophin loss causes a decrease in AChR ½ life, it would be expected that individual endplates would all lose similar amounts of receptors. Also, this rapid replacement is not a constant characteristic of fragmented junctions. Many fragmented junctions still retain high levels of BTX-1 (i.e. were classified as stable), suggesting that they suffered damage before, rather than during, the 10-day experimental period. Thus, these experiments suggest that the increased receptor replacement is caused by myofiber necrosis resulting from *mdx* muscle’s increased susceptibility to damage. We suggest the receptor replacement is not a primary effect of dystrophin loss, but a secondary effect resulting from dystrophic muscles having increased degeneration and regeneration. We performed further experiments to address whether myofiber damage itself was indeed causing the observed changes in NMJs.

### Dynamic junctions show morphological differences from stable *mdx* junctions

When morphological measurements of the NMJ were compared between dynamic and stable endplates of *mdx* mice, slight but significant differences were observed. At P38 the DI of dynamic endplates note more spread of the AChR at dynamic junctions (stable = 0.3725 ± 0.02, dynamic = 0.3069 ± 0.016, t(25) = 2.261, P = 0.0327, t-test). The same trend was evident at P66 (stable = 0.3738 ± 0.013, dynamic = 0.3098 ± 0.028, t(44) = 2.169, P = 0.0216, t-test). Additionally at P66, dynamic junctions had a lower receptor area than stable junctions (stable = 508.4 ± 34.21, dynamic = 304.4 ± 36.33, t(44) = 3.246, P = 0.0009, t-test). n is the number of pooled individual NMJs compared. No other attributes measured were significantly different between dynamic and stable junctions. At P66 almost all endplates were fragmented. These results suggest that not only can myofiber degeneration and regeneration cause a morphological shift from continuous to fragmented, but also that an already fragmented endplate can rearrange further following additional rounds of myofiber degeneration and regeneration. Continued remodeling may explain the increase in receptor dispersion in older *mdx* animals. While not measured, there were no obvious receptor clusters in *mdx* that were not covered by axon terminals. This corresponds well with our hypothesis that nerve terminal growth following myofiber regeneration is responsible for endplate remodeling. In other words, the nerve likely grows and branches along the myofiber, and directs new sites of receptor accumulation, in non-continuous islands. The regenerating muscle does not first deposit AChRs, which the nerve then innervates.

### Fiber damage at the endplate region of the muscle causes increased receptor replacement, in both *mdx* and WT animals

**Fig 4:**
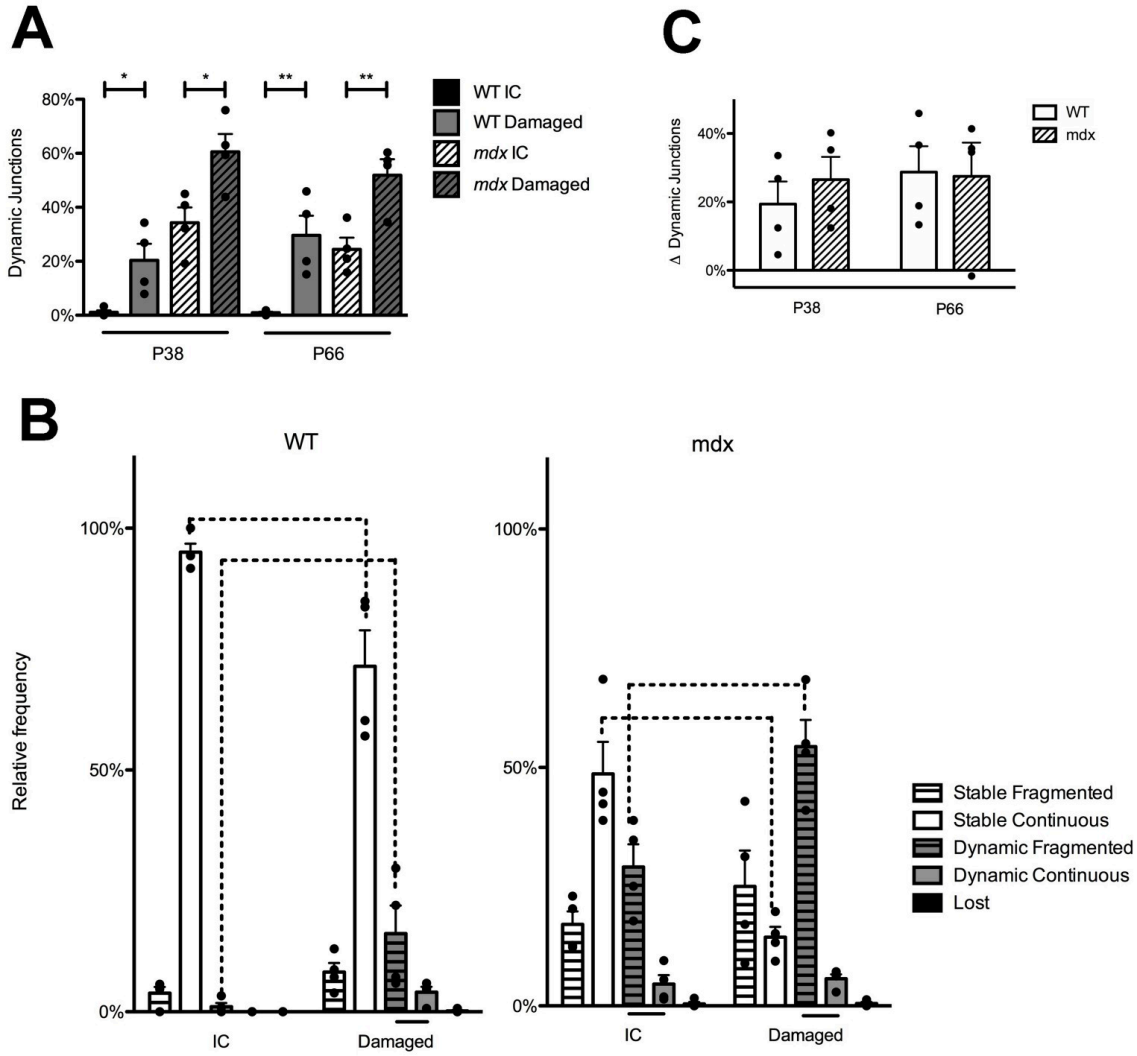
NMJ response to myofiber injury near the endplate in WT and *mdx* mice. (A) Receptor replacement following myofiber injury in WT and *mdx* STM. Both WT and *mdx* mice show increases in receptor replacement following damage as assessed by percentage of dynamic junctions. Asterisks denote significant differences (t-test, * P < 0.05, ** P < 0.001). (B) NMJ categorization following myofiber injury in P38 WT and *mdx*. Following muscle injury the proportion of dynamic, fragmented junctions is significantly increased and the proportion of stable, continuous junctions is decreased compared to ICs in both WT and *mdx* animals (2-Way ANOVA, Bonferroni post-hoc test, P < 0.001). Bars below the graphs indicate significant differences in the proportion of junctions within IC or Damaged groups (2-Way AVOVA, Bonferroni post-hoc test, P < 0.05), while dashed lines above the graphs indicate significant differences between the two groups (2-Way ANOVA, Bonferroni post-hoc test, P < 0.0001). Not all significant differences within genotypes are shown for sake of brevity. This information can be found S2 and S3 Tables. (C) Increases in receptor replacement following myofiber damage in WT and *mdx*. After damaging the STM, WT and *mdx* animals had similar increases in receptor replacement. This was true for both P38 and P66 animals. (t-test, n.s.). This result was interpreted to mean that WT and *mdx* animals have similar degenerative and regenerative responses to cut damage near the endplate, and that the magnitude of our injury was constant. Exact n can be found in text as t(df) = t-value. n = df + 2. n is the number of mice compared.

In order to test whether dynamic junctions are indeed a result of myofiber degeneration and regeneration, the same two-color BTX method was performed in conjunction with deliberate injury of the STM. Briefly, after the BTX-1 label was applied a superficial cut was made across the muscle with a scalpel near the endplate region to spur myofiber necrosis. The contralateral muscle was also labeled but not injured and used as an internal control (IC). When this was performed in P38 WT animals, the proportion of dynamic junctions in damaged muscles was 19.1x that of the IC (Fig 4A. P38 t(6) = 3.111, P = 0.0208. t-test). In P66 WT animals the proportion of dynamic junctions in damaged muscles was 31.6x as large as IC. (Fig 4A. P66, t(6) = 3.963, P = 0.0074, t-test). When deliberate injury was performed at the endplate area in P38 *mdx* the proportion of dynamic junctions in damaged muscle was 1.7x as large as that of the IC, and at P66 the proportion of dynamic junctions in damaged muscle was 2.1x as large as the IC (Fig 4A. P38 t(6) = 3.013, P = 0.0236; P66 t(6) = 3.772, P = 0.0093, t-test). This shows that myofiber damage near the endplate area is sufficient to induce increases in AChR replacement in both WT and *mdx* mice.

In order to see if this muscle damage-induced receptor replacement had an effect on the morphology of junctions, dynamic fragmented and dynamic, continuous junctions were compared within injured muscles. In other words, we investigated whether dynamic junctions were preferentially fragmented in the muscle injury paradigm. P38 animals were again used because at this time in *mdx* there is a population of both fragmented and continuous junctions, while after P66 most *mdx* junctions are fragmented. Therefore, in P38 animals any differences in dynamic junction morphology caused by myofiber damage could be noted. Within damaged P38 *mdx* STMs the proportion of dynamic, fragmented junctions was 9.6x that of the proportion of dynamic, continuous junctions (Fig 4B. t(15) = 8.279, P < 0.001, 2-Way ANOVA, Bonferroni post-hoc test). Within damaged WT muscles the proportion of dynamic, fragmented junctions was 4.5x that of the proportion of dynamic, continuous junctions (Fig 4B. t(15) = 2.725, P < 0.05, 2-Way ANOVA, Bonferroni post-hoc test). The prevalence of dynamic, fragmented junctions over continuous within injured muscles suggests myofiber necrosis and regeneration is correlated with NMJ remodeling. A full account of NMJ type within the groups (i.e dynamic, continuous vs stable, fragmented in WT damaged muscles, etc) is given in S2 and S3 Tables.

The hypothesis that myofiber degeneration and regeneration cycles cause increases in receptor replacement and is correlated to NMJ remodeling is further substantiated when the ICs are investigated. Within *mdx* ICs, the proportion of dynamic, fragmented junctions was 6.4x that of the proportion of continuous junctions that were also dynamic (Fig 4B. t(15) = 4.179, P < 0.001, 2-Way ANOVA, Bonferroni post-hoc test). This trend was not noted for WT IC muscles. In non-injured WT muscles, it is unlikely that the muscles have been damaged naturally, whereas *mdx* muscles begin to show signs of myofiber degeneration around 3 weeks of age [31–33]. Again, because the receptor replacement in WT ICs is so small, it is possible any NMJ remodeling correlated to replacement may have not been appreciated. These results suggest that muscle damage does cause increases in receptor replacement, and that it is correlated to NMJ remodeling.

Additional evidence that myofiber damage causes NMJ remodeling is found when damaged muscles are compared directly to ICs. Following deliberate myofiber injury in P38 WT muscles the proportion of dynamic, fragmented junctions was 15.4x that of IC dynamic, fragmented junctions (Fig 4B. WT, t(6) = 3.398, P < 0.01, 2-Way ANOVA, Bonferroni post-hoc test). In damaged P38 *mdx* muscles the proportion of dynamic, fragmented junctions was 1.9x that of IC dynamic, fragmented junction (Fig 4B. *mdx* t(6) = 4.289, P < 0.001, 2-Way ANOVA, Bonferroni post-hoc test). There was also a corresponding loss of stable, continuous junctions in damaged muscles compared to ICs for both genotypes (Fig 4B. WT, t(6) = 5.299, P < 0.001, *mdx* t(6) = 5.820, P < 0.001, 2-Way ANOVA, Bonferroni post-hoc test). This investigation suggests that myofiber necrosis and regeneration at the endplate region is sufficient to shift endplate morphology from continuous to fragmented.

To investigate whether WT and *mdx* muscles responded to endplate damage in a similar fashion, the increase in the proportion of dynamic junctions in injured muscles compared to ICs was calculated. WT and *mdx* muscles showed no difference in response to injury at P38 or P66 (Fig 4C. n.s. t-test). This indicates that baseline replacement due to exogenous injury is the same between WT and *mdx* mice, and that the magnitude of the injury was the same. As the receptor replacement response due to myofiber damage at the endplate area of WT mice mimics that seen in uninjured *mdx* mice, we believe it likely that degeneration and regeneration of the muscle is the cause of both receptor replacement and remodeling.

### The motor axon is needed to restructure the postsynaptic endplate

**Fig 5:**
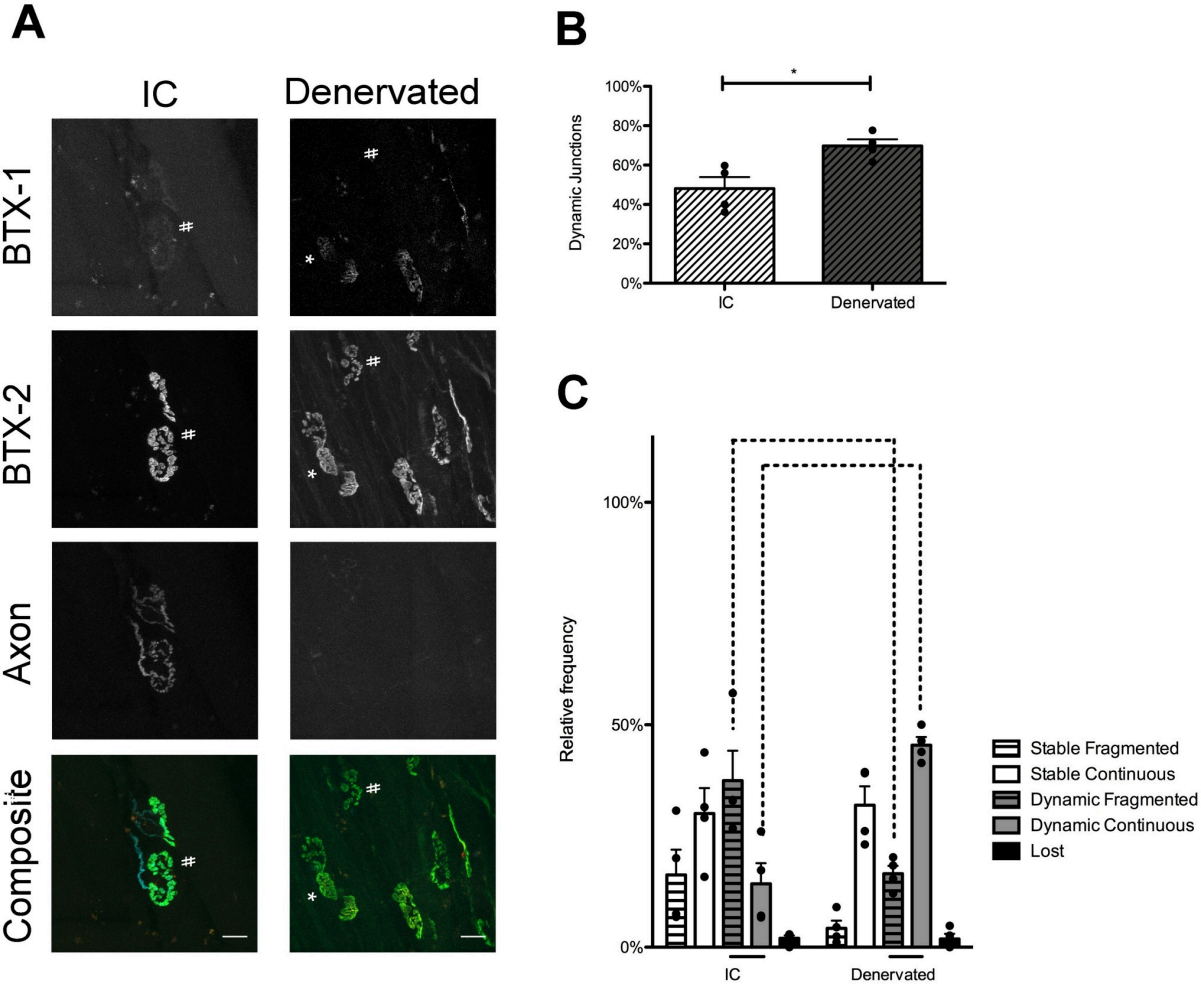
Innervation is required for NMJ remodeling in P38 *mdx* mice. (A) Confocal maximum intensity projection of P38 *mdx* innervated (IC) and denervated STMs. “#” denotes dynamic, fragmented junctions. Asterisks denotes dynamic, continuous junctions. In the composite image BTX-1 has been pseudocolored red, BTX-2 green, and motor axon cyan. Note that in the denervated muscle there is no motor axon labeling in the maximum intensity projections. This indicates motor axon loss and no regeneration during the 10-day recovery period. Additionally, both the IC and denervated collections have NMJs with little or no BTX-1 label. This indicates AChR replacement attributed to myofiber degeneration/regeneration in both NMJs with and without innervation Scale bar = 20 μm. (B) Receptor replacement in P38 *mdx* following denervation. Receptor replacement, as assessed by dynamic junctions, is elevated in P38 *mdx* denervated muscles as compared to IC (t-test, P < 0.05). (C) NMJ categorization in P38 *mdx* mice after denervation. Following denervation, the proportion of dynamic, fragmented junctions is significantly decreased, and the proportion of dynamic, continuous junctions is decreased compared to non-denervated IC (2-Way ANOVA, Bonferroni post-hoc test, P < 0.001). Bars below the graphs indicate significant differences in the proportion of junctions within IC or denervated groups (2-Way AVOVA, Bonferroni post-hoc test, P < 0.05), while bars above the graphs indicate significant differences between the two groups (2-Way ANOVA, Bonferroni post-hoc test, P < 0.0001). Not all significant differences within genotypes are shown for sake of brevity. This information can be found in S4 Table. Exact n can be found in text as t(df) = t-value. n = df + 2. n is number of mice compared.

Above, we have shown that muscular dystrophy in *mdx* mice causes increases in AChR replacement. The increase is due to myofiber degeneration and regeneration at the synaptic site and is correlated with abnormal endplate morphology. We next investigated the role that the innervating motor axon plays in receptor dynamics and endplate remodeling in *mdx* mice. It has previously been suggested in *mdx* mice that the nerve is necessary to restructure the endplate upon muscle cell regeneration [34]. However, in this experiment whether or not the endplate area of the muscle was injured could not be directly assessed. To investigate the role of innervation in remodeling specifically regenerated NMJs, the two-color BTX approach was again utilized, but the nerve that innervates a single STM (accessory nerve) was severed at the time of the first BTX exposure. After the 10-day convalescent period the nerve had not regrown to the endplate region and no NMJs were innervated. The contralateral muscle was left innervated as an IC. The procedure was initiated in P28 mice. Again, at the endpoint of this experiment (P38) there are still a large number of junctions that are not fragmented in *mdx* mice, so we are able to record any changes in the proportion of fragmented or continuous junctions with and without the nerve. Receptor replacement in denervated *mdx* muscles was elevated from ICs (Fig 5B. t(6) = 3.227, P = 0.018, t-test). This was not surprising as denervation and/or blocking muscle contractions is known to promote AChR replacement [28, 35]. However, in comparison to innervated muscles, denervated P38 *mdx* STMs had an increase in the number of dynamic, continuous junctions (Fig 5C. t(6) = 5.484, P < 0.001, 2-Way ANOVA, Bonferroni post-hoc test) and a corresponding decrease in dynamic, fragmented junctions (Fig 5C. t(6) = 3.684, P < 0.01, 2-Way ANOVA, Bonferroni post-hoc test).

## Discussion

This study provides strong evidence that NMJ fragmentation in *mdx* muscles is initiated by bouts of myofiber degeneration and regeneration, and that the innervating motor axon terminal must be present during fiber regeneration to instruct NMJ remodeling. The morphological abnormalities that are evident in mouse *mdx* NMJs also occur in the more clinically relevant canine GRMD model and the same processes that instruct remodeling mice likely also occur in GRMD. This suggests that NMJ remodeling is a common consequence of muscular dystrophy in all animals, including human DMD patients. We also provide evidence that the remodeling progresses with age, as a result of ongoing degenerative and regenerative events that occur in STM of *mdx* mice well into adulthood. Indeed, we do not think that many of the changes seen in dystrophy, such as increase in Junctional Area, are due to increases in muscle fiber diameter. As muscle fibers grow, the receptor aggregate upon them also increases in size. One could postulate that the increase in Junctional Area of *mdx* junctions is a function of increased dystrophic muscle fiber size, possibly due to hypertrophy. This hypothesis does not hold under scrutiny for one decisive reason. While variation in muscle fiber cross sectional area is higher in *mdx*, the average myofiber cross sectional area is no different between WT and *mdx* animals [36, 37]. This shows that the size of a dystrophic junction grows at a rate that is not mimicked by the myofiber cross sectional area. Observationally, much of the increase in dystrophic NMJ size occur along the length of the myofiber (see Figs 1A and 2A), though a direct measurement of the axis of growth was not made.

While many have postulated that the NMJ changes in *mdx* animals are due to myofiber damage, there has been little direct investigation of the link between these phenomena. Other investigators have correlated central chains of nuclei within a myofiber to fragmented junctions, but it is unclear if central nuclei are a marker for recent myofiber regeneration [9]. Historically, myofibers with nuclei in the center of the cytoplasm instead of the periphery have been used to identify regenerated muscles [38]. Unfortunately, this method of investigating regeneration has its drawbacks. For one, the time course over which myonuclei move from the center to the periphery of the fiber may be very long and not indicate truly recent regeneration [13]. Using the in vivo two-color BTX method it is possible to identify recently regenerated muscle fibers and to categorize their NMJs by morphology. The two-color method is appealing for studying myofiber regeneration at the endplate as it does not rely on myonucleur location. This evidence suggests dynamic junctions indicate a recent myofiber degeneration and regeneration cycle at the NMJ.

Using the two-color BTX method also provided evidence that myofiber degeneration and regeneration is continuously elevated in *mdx* STM from P38 to P450. This finding contradicts previous reports that have described the pathology of *mdx* muscles as transitioning from an early necrotic stage at 3 weeks, to a regenerative state that closes at 12 weeks [17], although it must be mentioned that the STM was not investigated in these reports. Using tritiated thymidine and investigating satellite cell nucleus incorporation into myofibers, an elevated level of myofiber regeneration in *mdx* was shown to be restricted to an acute period from P24 to P50. A chronic phase of myopathy follows with a much lower level of labeled nuclei [39]. A similar observation of an early crisis period in *mdx* mice has been described by researchers via embryonic myosin expression and histological methods [17, 33]. However other reports suggest that myofiber damage may be still be vastly elevated past this crisis period [36, 40]. The results of the two-color BTX investigation hold with the later conclusion. If the loss of the first BTX label is indeed due to myofiber necrosis at the synaptic site, our results above show that the degenerative and regenerative cycle of muscle fibers extends well into the adult life of *mdx* STMs.

Furthermore, the results show it is unlikely that the dystrophin loss itself causes increased receptor replacement, but such replacement is rather due to myofiber damage resulting from the loss of the protein. Deliberate damage to muscle fibers in WT mice resulted in similar receptor replacement and NMJ remodeling as seen in *mdx* muscles. While there are reports of clustering abnormalities in myotubes in vitro lacking dystrophin [18], in living *mdx* animals AChRs clusters develop normally into a pretzel shaped morphology early in life. It is only after the onset of myofiber necrosis at P21 that remodeling begins to occur. This suggests that myofiber damage is causative in NMJ remodeling in *mdx* mice. It has been shown that in other mouse models NMJ remodeling can occur in without myofiber damage [41, 42]. However, deliberate injury to the fibers near their endplates increased NMJ remodeling in *mdx* muscles.

The ½ life of AChRs in healthy, innervated NMJs is about 14 days [28]. It has also been shown that there are different pools of AChR with distinct ½ lives, the shortest being about 1 day [43]. However, even when a similar in vivo two-color BTX technique was used in NMJs where metabolic receptor turnover was increased through long term denervation [43], the catastrophic loss of the first BTX label was not observed, as we see in dynamic junctions. This we believe shows the reliability of our measurements, and that dynamic junctions do indeed represent myofiber degeneration and regeneration. Again, while some loss of BTX-1 would be expected even in WT animals, the exaggerated, almost complete loss of receptors from dystrophic muscles over a 10-day period is abnormal. It must however be noted that in the reported experimental procedure fibers that had been deliberately damaged could not be relocated when imaged. We are therefore unable to definitely link myofiber damage and dynamic junctions. Previous investigation, however, has shown that when deliberately damaged muscles are imaged over time NMJs become dynamic [19]. It is possible that receptor replacement in not occurring on damaged myofibers but on fibers subjected to disuses or an inflammatory response. An effort, of course, was made to recover muscle fibers in the area of damage, but this cannot be guaranteed. Additionally, experiments are currently ongoing using the dye Procion orange in vivo to label damaged myofibers, as Evan’s Blue Dye was ineffective is our whole mount muscle preparations necessary for imaging and NMJ categorization.

Increased replacement has been observed at NMJs whose receptors have been blocked by a sufficiently high dose of BTX to cause any remaining neuromuscular transmission to fall below threshold for generation of muscle fiber action potentials. However, in these experiments, a sufficiently low concentration of BTX known to not accelerate the replacement of receptors was used to allow continued transmission, and to not perturb morphology [28].

The STM was chosen for examination in these experiments, largely because of its accessibility for BTX application and for in vivo labeling. It is however clear that different muscles in muscular dystrophy are affected to different degrees. For example, one of the muscles most affected in murine dystrophy is the diaphragm [44, 45], while the extraocular muscles are spared [46]. The diaphragm exhibits signs of constant degeneration and subsequent fibrosis and adipose tissue deposition unlike many other muscles studied in the mouse. This likely is due the amount of activation (and damage) to which the diaphragm is subjected via contractions during respiration. A similar phenomenon may be occurring in the STM of *mdx* mice. It is documented that the trunk and neck muscles of neonatal GRMD dogs are more severely affected than appendicular muscle groups [47], likely because they are postural muscles and they are used more constantly than muscles involved with movement, and therefore subjected to increased contraction induced damage. This was noted in neonatal GRMD, but atrophy of the truncal muscles has been noted in older animals as well [48]. The STM has not been typically investigated in dystrophy. It would be interesting to do a similar two-color BTX experiments in muscles that have been proposed to undergo a crisis period of degeneration, namely the tibialis anterior or soleus limb muscles. Such experiments would be difficult in diaphragm, due to accessibility issues. Investigations of the tibialis anterior are currently underway.

Previous studies have shown that the innervating motor axon terminal plays a role in remodeling of the AChRs at the synapse after muscle injury [19] and some experiments have suggested this in the case of dystrophy [34]. This investigation was able to examine individual myofibers and synaptic sites that have recently undergone replacement due to myofiber necrosis. In the absence of the nerve, the receptors are still lost from the membrane, but upon regeneration the morphology is continuous. It also suggests that *mdx* muscle damage can occur without transmission induced contraction, possibly due to preexisting fenestrations in the sarcolemma. Excepting denervation-induced fibrillation [49, 50], it is unlikely that denervated muscles experience much contractile activity. Another explanation of BTX-1 loss in denervated STM is that prior to denervation, the muscle fiber was damaged, but had not begun the process of necrosis. We hypothesize that in *mdx* mice, myofiber damage caused by lack of dystrophin leads to degeneration and regeneration of the muscle fiber and this is the first step in the cascade of events that leads to NMJ rearrangement. During regeneration, the innervating motor neuron, which remains on the basal lamina at the synaptic site of the damaged myofiber, sends out “sproutlets” and varicosities to search for the regenerating fiber [19]. As the fiber regenerates, new AChR are inserted into the sarcolemma at areas that are contacted by these varicosities and sproutlets. This causes a fragmented appearance on the postsynaptic AChR aggregate. If the motor axon is not present, when the myofiber regenerates, the AChR aggregate will remain in its original morphology. That is to say if the aggregate was previously continuous it will remain continuous upon myofiber regeneration. We show that the innervating motor axon is responsible for NMJ fragmentation, a hallmark of the dystrophic endplate. Without direction from the axon, NMJ remodeling does not occur. It is probable that factors like agrin released from the sproutlets result in the fragmented distribution of AChR.

A confounding attribute to the denervation experiments is that in the absence of innervation, myofibers undergo atrophy [51, 52]. The increases in the number of continuous junctions may be a result of myofiber collapse. It is possible that the junctions do in fact restructure themselves morphologically even in the absence of neural input, but upon subsequent myofiber atrophy the fragments connect with each other. From the parameters this report and others use to define fragmented (i.e. having 5 or more non-continuous clusters of AChR) versus continuous junctions, this remodeling might not have been realized. Whether the sparing of morphological change has any physiological consequence, remains to be investigated.

Neuromuscular transmission properties of remodeled endplates in dystrophy should be closely investigated. There are a number of published studies that have researched the functionality and electrophysiology of dystrophic NMJs. However, the results are often contradictory, especially when mEPP amplitude and variability are investigated [9, 53–55]. This could be due to the differences in experimental methods, or perhaps to the variability of the disease state and its progression. Researchers have shown that in aged NMJs, which have a similar morphology to dystrophic and damaged NMJs, there is little difference between normal and remodeled junction physiology [56]. In fact, the fragmented morphology may increase the efficacy of transmission. Whether or not this is the same for dystrophy remains to be clearly elucidated but some experiments on the physiology of such junctions indicate an absence of major defects [9] while others show obvious deficiencies in neuromuscular transmission [57]. However, the current study shows the changes in junction morphology are progressive, in that remodeled junctions can likely further remodel. It is possible that significant changes in the physiology of neuromuscular transmission do appear in the later stages of the disease. Interestingly, in DMD patients and clinically relevant animal models, such as GRMD, there is evidence of neuromuscular transmission deficiencies [26, 58, 59].

This study and others have shown that changes to the structure of the NMJ occur rapidly and irreversibly. Present treatments for DMD do not commonly begin until after the onset of the disease at which time changes to the morphology of the NMJ have already occurred. We believe it is important to study the remodeling of the NMJ as mitigation of this remodeling might alter the progression of the disease. This would especially be the case if remodeled junctions are physiologically deficient. This study demonstrates that the sequence of events that lead to altered NMJs is dependent upon the motor neuron but initiated by muscle damage. It also indicated that NMJ abnormalities are common to animal models without dystrophin expression at the muscle membrane, and likely applies to affected DMD humans as well. We suggest that an additional component to treatment of the disease besides sparing of muscle degeneration would be altering the changes that occur in muscle innervation.

## Supporting information

S1 Fig Identification of tSCs. Confocal maximum intensity projection images of a P66 WT mouse NMJ. Arrowheads indicate S100:eGFP positive cells with nuclei in the fluorescent somata near the AChR rich endplate. Not all cells are indicated. These were considered tSCs for tSC counts. Composite image in red-AChR, green-S100:eGFP positive cells, gray-nuclei. Scale bar = 20 μm^2^.
(TIF)

S2 Fig Motor axon varicosities in GRMD NMJs. Confocal maximum intensity projection images of an adult GRMD dog NMJ. Arrows indicate bulbous varicosities of motor axons that synapse on the AChR endplate. Composite image in red-AChR, cyan-motor axon labeled with neurofilament and synaptic vesicle protein antibodies. Scale bar = 20 μm^2^.
(TIF)

S3 Fig NMJ Characterization and Reproducibility (40X). Maximum intensity projection images of P38 *mdx* NMJs following in vivo two-color BTX method. In composite BTX-1 is pseudocolored red and BTX-2 green. Yellow indicates strong colocalization. Black arrows denote stable, continuous junctions. Black arrow heads denote stable, fragmented junctions. White arrow heads denote dynamic, fragmented junctions. Asterisks denote lost junctions. Please note the presence of both dynamic and stable junctions in proximity to each other. This shows that receptor replacement occurs at individual junctions independent of neighboring endplates. This suggest that the absence of dystrophin does not simply shorten AChR ½ life and that catastrophic receptor loss and replacement are likely due to myofiber degeneration and regeneration. Scale bar = 20 μm.
(TIF)

S4 Fig NMJ Characterization and Reproducibility (20X). Maximum intensity projection images of P38 and P66, *mdx* and WT NMJs following in vivo two-color BTX method. In composite BTX-1 is pseudocolored red and BTX-2 green. White arrows show examples of dynamic, continuous junctions. White arrow heads denote dynamic, fragmented junctions. Scale bar = 50 μm.
(TIF)

S1 Table Chart of significant differences in NMJ categorization following in vivo two-color BTX experiments (P38 WT and *mdx*). Comparisons within genotypes. 2-Way ANOVA with Bonferroni post-hoc test. Red boxes indicate redundancy. Black boxes indicate comparisons of the same categories. * P < 0.05. ** P < 0.01. *** P < 0.001.
(PDF)

S2 Table Chart of significant differences in NMJ categorization following in vivo two-color BTX experiments (P38 WT myofiber damage and IC). Comparisons within groups. 2-Way ANOVA with Bonferroni post-hoc test. Red boxes indicate redundancy. Black boxes indicate comparisons of the same categories. * P < 0.05. ** P < 0.01. *** P < 0.001.
(PDF)

S3 Table Chart of significant differences in NMJ categorization following in vivo two-color BTX experiments (P38 *mdx* myofiber damage and IC). Comparisons within groups. 2-Way ANOVA with Bonferroni post-hoc test. Red boxes indicate redundancy. Black boxes indicate comparisons of the same categories. * P < 0.05. ** P < 0.01. *** P < 0.001.
(PDF)

S4 Table Chart of significant differences in NMJ categorization following in vivo two-color BTX experiments (P38 *mdx* denervation and IC). Comparisons within groups. 2-Way ANOVA with Bonferroni post-hoc test. Red boxes indicate redundancy. Black boxes indicate comparisons of the same categories. * P < 0.05. ** P < 0.01. *** P < 0.001.
(PDF)

S1 Movie 3D rotation of P66 *mdx* NMJ. 3D maximum intensity projection of confocal stack. 0.5 μm gap between collection slices interpolated via Image J 3D Projection tool. BTX-1 pseudocolored red. BTX-2 pseudo colored green. DAPI nuclear label pseudocolored gray. Note the occurrence of a central chain of nuclei running within the myofiber directly under the dynamic, fragmented junction. This is the same junction featured in Fig 3A.
(M4V)
