# Supplementary figures and images for: Cycles of myofiber degeneration and regeneration lead to remodeling of the neuromuscular junction in two mammalian models of Duchenne muscular dystrophy
Source: PLoS One. 2018 Oct 31;13(10):e0205926. doi: 10.1371/journal.pone.0205926 (PMC6209224; doi:10.1371/journal.pone.0205926)

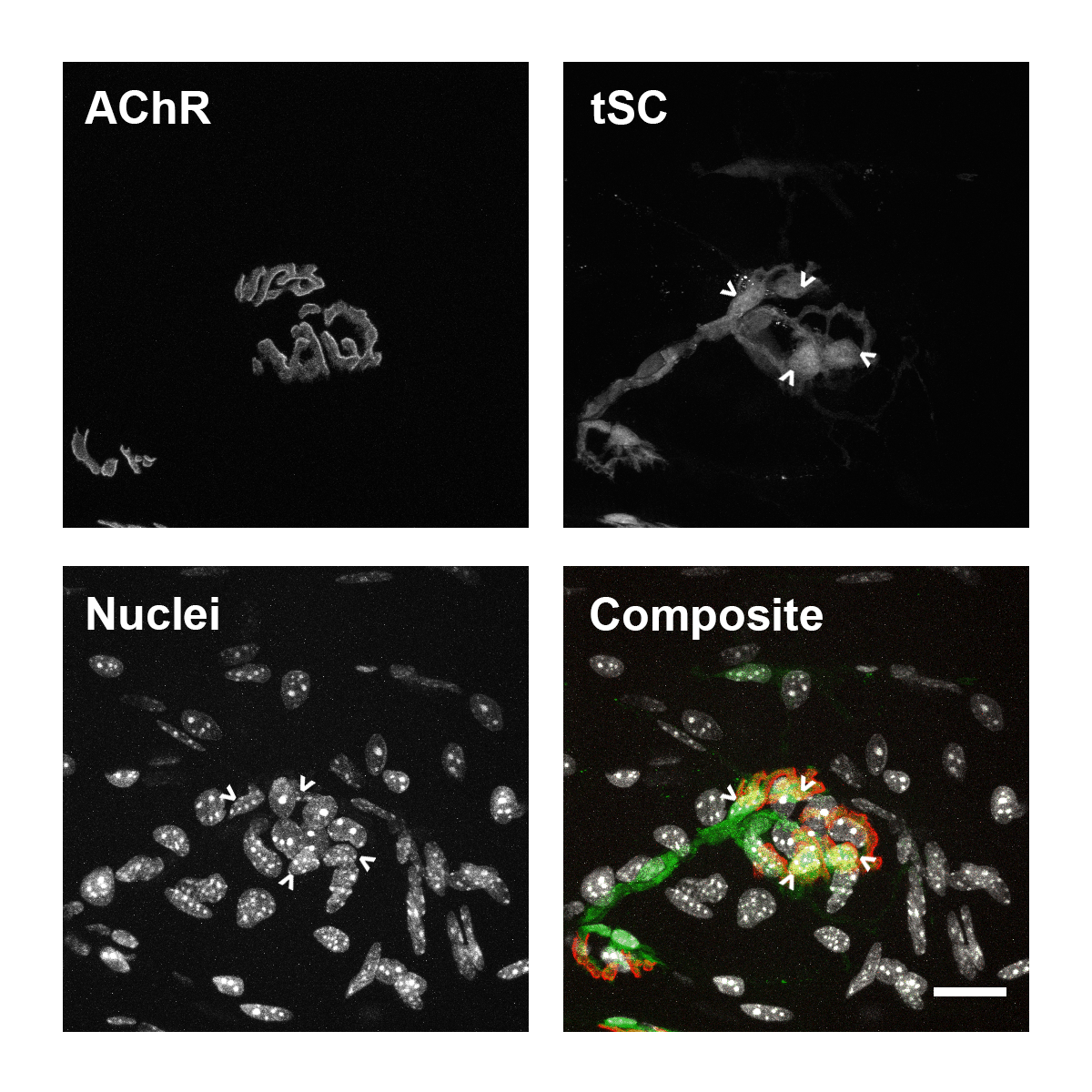

Supplement: S1 Fig — Confocal maximum intensity projection images of a P66 WT mouse NMJ. Arrowheads indicate S100:eGFP positive cells with nuclei in the fluorescent somata near the AChR rich endplate. Not all cells are indicated. These were considered tSCs for tSC counts. Composite image in red-AChR, green-S100:eGFP positive cells, gray-nuclei. Scale bar = 20 μm2. (TIF) [file pone.0205926.s001.tif]

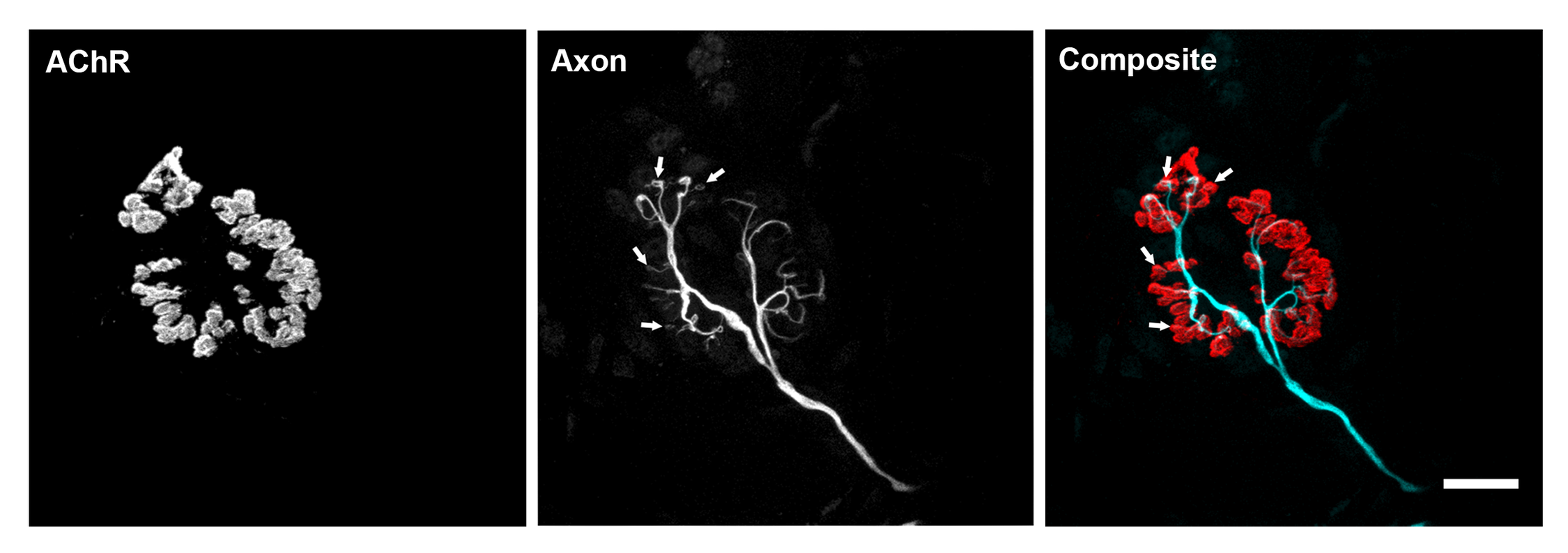

Supplement: S2 Fig — Confocal maximum intensity projection images of an adult GRMD dog NMJ. Arrows indicate bulbous varicosities of motor axons that synapse on the AChR endplate. Composite image in red-AChR, cyan-motor axon labeled with neurofilament and synaptic vesicle protein antibodies. Scale bar = 20 μm2. (TIF) [file pone.0205926.s002.tif]

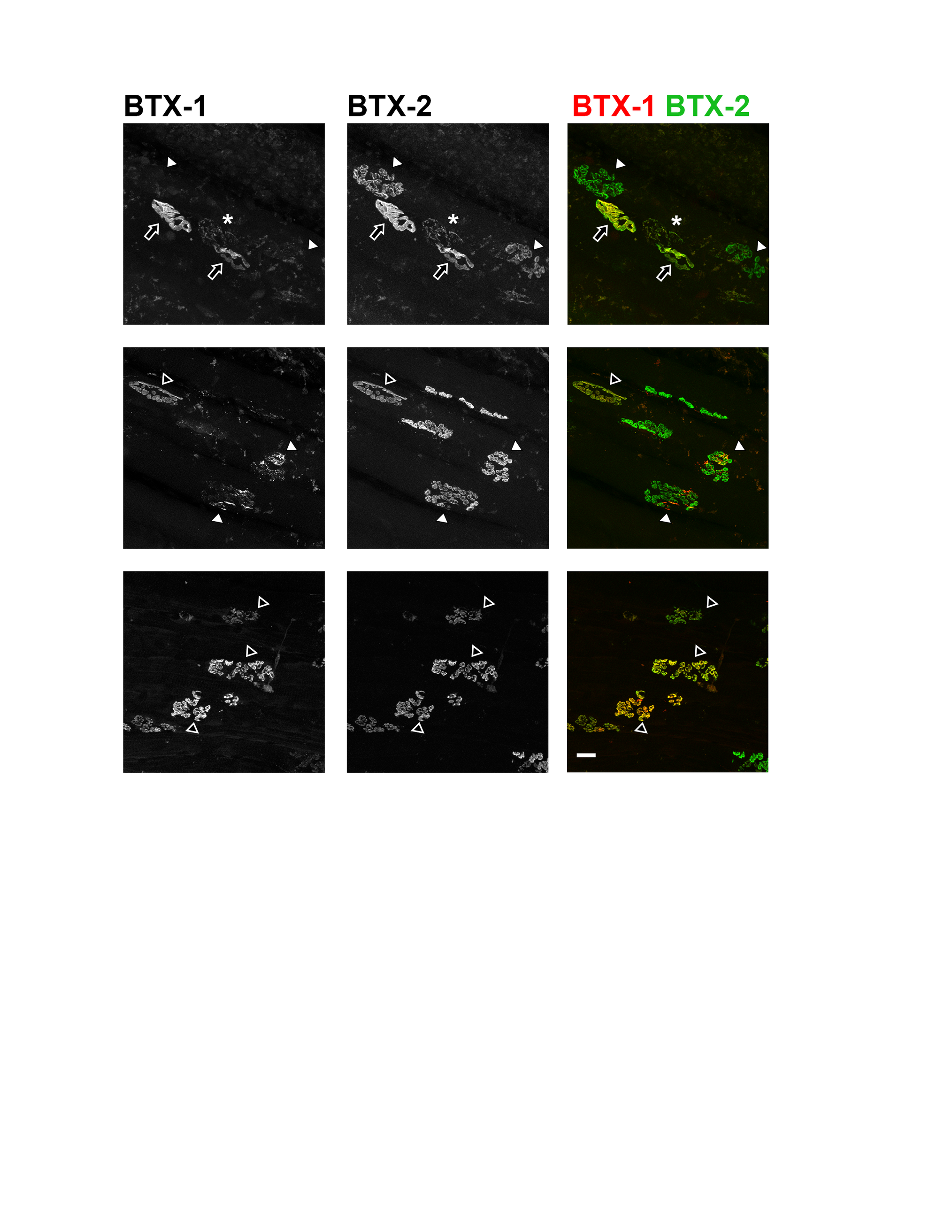

Supplement: S3 Fig — Maximum intensity projection images of P38 mdx NMJs following in vivo two-color BTX method. In composite BTX-1 is pseudocolored red and BTX-2 green. Yellow indicates strong colocalization. Black arrows denote stable, continuous junctions. Black arrow heads denote stable, fragmented junctions. White arrow heads denote dynamic, fragmented junctions. Asterisks denote lost junctions. Please note the presence of both dynamic and stable junctions in proximity to each other. This shows that receptor replacement occurs at individual junctions independent of neighboring endplates. This suggest that the absence of dystrophin does not simply shorten AChR ½ life and that catastrophic receptor loss and replacement are likely due to myofiber degeneration and regeneration. Scale bar = 20 μm. (TIF) [file pone.0205926.s003.tif]

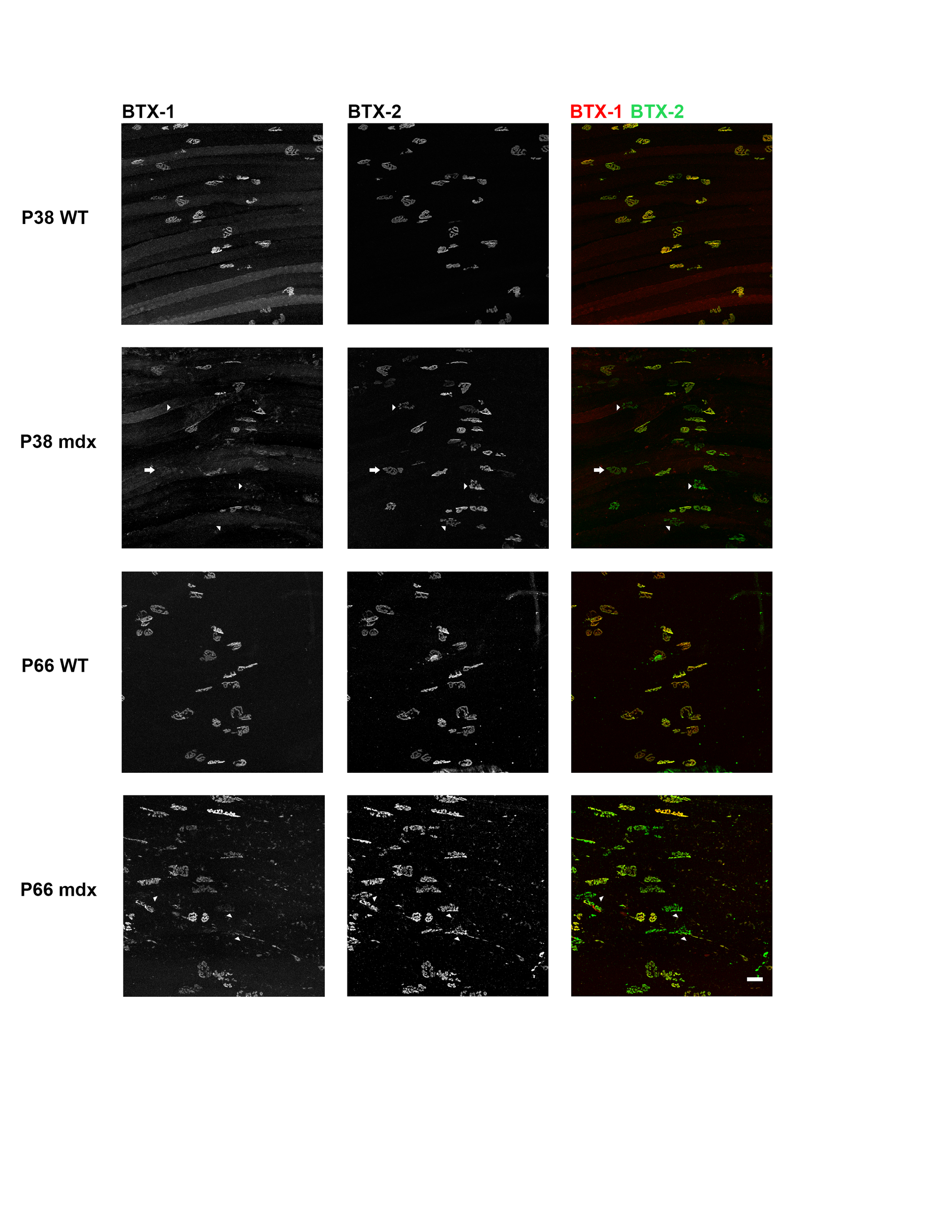

Supplement: S4 Fig — Maximum intensity projection images of P38 and P66, mdx and WT NMJs following in vivo two-color BTX method. In composite BTX-1 is pseudocolored red and BTX-2 green. White arrows show examples of dynamic, continuous junctions. White arrow heads denote dynamic, fragmented junctions. Scale bar = 50 μm. (TIF) [file pone.0205926.s004.tif]
