# Supplementary material for: Cycles of myofiber degeneration and regeneration lead to remodeling of the neuromuscular junction in two mammalian models of Duchenne muscular dystrophy
Source: PLoS One. 2018 Oct 31;13(10):e0205926. doi: 10.1371/journal.pone.0205926 (PMC6209224; doi:10.1371/journal.pone.0205926)
Supplement: S1 Table — Comparisons within genotypes. 2-Way ANOVA with Bonferroni post-hoc test. Red boxes indicate redundancy. Black boxes indicate comparisons of the same categories. * P < 0.05. ** P < 0.01. *** P < 0.001. (PDF) [file pone.0205926.s005.pdf]

WT		Stable		Dynamic		
		Continuous	Fragmented	Continuous	Fragmented	Lost
Stable	Continuous		***	***	***	***
	Fragmented			ns	ns	ns
Dynamic	Continuous				ns	ns
	Fragmented					ns

mdx		Stable		Dynamic		
		Continuous	Fragmented	Continuous	Fragmented	Lost
Stable	Continuous		ns	***	ns	***
	Fragmented			***	ns	***
Dynamic	Continuous				***	ns
	Fragmented					***
