# Supplementary material for: Cycles of myofiber degeneration and regeneration lead to remodeling of the neuromuscular junction in two mammalian models of Duchenne muscular dystrophy
Source: PLoS One. 2018 Oct 31;13(10):e0205926. doi: 10.1371/journal.pone.0205926 (PMC6209224; doi:10.1371/journal.pone.0205926)
Supplement: S3 Table — Comparisons within groups. 2-Way ANOVA with Bonferroni post-hoc test. Red boxes indicate redundancy. Black boxes indicate comparisons of the same categories. * P < 0.05. ** P < 0.01. *** P < 0.001. (PDF) [file pone.0205926.s007.pdf]

mdx Internal Control		Stable		Dynamic		Lost
		Continuous	Fragmented	Continuous	Fragmented	
Stable	Continuous		***	***	**	***
	Fragmented			ns	ns	*
Dynamic	Continuous				***	ns
	Fragmented					***

mdx Endplate Damage		Stable		Dynamic		Lost
		Continuous	Fragmented	Continuous	Fragmented	
Stable	Continuous		ns	ns	***	*
	Fragmented			**	***	***
Dynamic	Continuous				***	ns
	Fragmented					***
